# Case of Complete Remission After Volumetric Modulated Arc Therapy to Primary Tumor Alone in Locally Advanced Anal Canal Cancer With Active AIDS and Low CD4 Cell Count: Longest Survival in History?

**DOI:** 10.7759/cureus.9093

**Published:** 2020-07-09

**Authors:** Ming Pan

**Affiliations:** 1 Radiation Oncology, Windsor Regional Hospital Cancer Program, Windsor, CAN

**Keywords:** human immunodeficiency virus, acquired immune deficiency syndrome, anal canal cancer, combined modality treatment, volumetric modulated arc therapy, colostomy

## Abstract

The incidence of anal canal cancer (AC) is increased in HIV-positive individuals and is often associated with poor prognosis. High viral load and low CD4 cell count have long been considered relative contraindications for combined modality treatment (CMT) with concurrent chemotherapy and external beam radiation treatment (EBRT) for AC due to severe toxicities. EBRT alone is quite often considered as palliative treatment in nature. We report a case of complete remission (CR) of locally advanced anal canal squamous cell carcinoma (ACSCC) cured after volumetric modulated arc therapy (VMAT) to the primary tumor alone in a 62-year-old male with a 30-year history of AIDS, characterized by an HIV viral load over one million and low CD4 cell count around 100 mm^-3^. VMAT achieved excellent long-term local control of AC and good quality of life (QoL) of the patient without severe toxicity that requires diverting colostomy.

## Introduction

AIDS is diagnosed when there is a demonstration of HIV infection, the number of CD4 cells falls below 200 mm^-3^, or when patients develop one or more opportunistic infections and specific cancers, that is, the two major AIDS-defining cancers being Kaposi sarcoma and non-Hodgkin lymphoma, regardless of their T cell CD4 counts. Without treatment, life expectancy is approximately three years. However, with certain opportunistic infections, survival may be less than one year. The incidence of anal canal cancer (AC) is increased in HIV-positive individuals. The relative risk of AC in people with AIDS is 31.7 (95% CI 11.6 to 69.2). Although not an AIDS-defining diagnosis, the frequent occurrence of AC in HIV-positive patients warrants specific consideration [1-3].

Survival beyond two years, without colostomy, is possible with standard high dose (at least 45-Gy) CMT for patients who have stage T1 or T2 cancer, with good performance status and without AIDS. However, high viral load and low CD4 count have long been considered relative contraindications of combined modality treatment (CMT) for AC due to severe toxicities, despite the fact that good results have been consistently reported to use CMT for HIV-negative AC patients [4]. For patients with evidence of progressive HIV disease or low CD4 counts, palliative low-dose external beam radiation treatment (EBRT) (such as 30-Gy in 10 fractions) is often required in some institutions to control pain, bleeding, and infection while reducing toxicity, but others found no such need to reduce the EBRT or chemotherapy dose in HIV-positive AC patients without AIDS [4-10]. The numbers of patients in these reports are very small. It is almost impossible to conduct phase III randomized controlled trials (RCT) to test whether potentially curative treatment without severe toxicities is possible in these rare subgroups of AC patients.

## Case presentation

**Figure 1:**
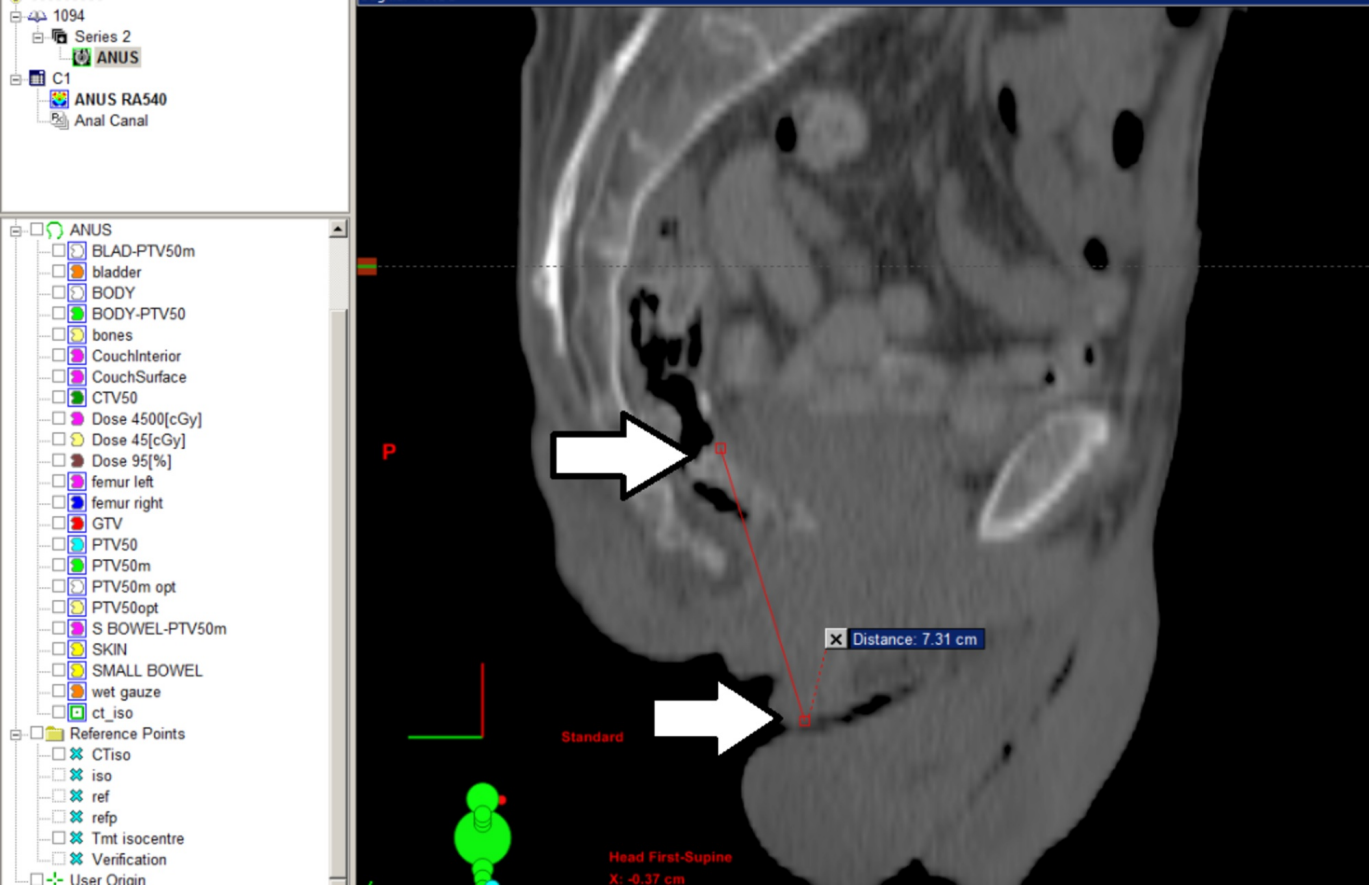
CT imaging showing large tumor over 7 cm in length (white arrows), involving peri-anal skin, the entire anal canal, and lower rectum CT, computed tomography

A 62-year-old man presented with peri-anal and anal canal mass. Biopsy confirmed anal canal squamous cell carcinoma (ACSCC) involving the peri-anal skin and the anal canal on January 6, 2014. Tumor markers showed positive CK and p16, but negative HPV. CT scan of the chest, abdomen, and pelvis did not show any evidence of lymph node or distant metastases. The thickened mass from anorectal junction to anus measured 7 cm in length (Figure 1). He was staged as T3N0M0 according to the American Joint Committee on Cancer (AJCC) 7th Edition [11].

He had been diagnosed with AIDS after being tested HIV-positive in 1986. He had a long history of noncompliance with highly active antiretroviral therapy (HAART) and had a very high viral load, over one million, and baseline CD4 cell count around 100 mm^-3^ for the immediate two years before presentation (range 105-128 mm^-3^). He had multiple infections with abscess treated with local excision, drainage, and antibiotics. He also had multiple recurrent condyloma removed from the peri-anal area.

At the time of his AC diagnosis, his HIV viral load was 1,081,193 and CD4 was only 105 mm^-3^. CBC showed WBC at 4.6 x 10^9^/L, RBC at 4.74 x 10^12^/L, platelets at 153 x 10^9^/L, hemoglobin at 141 g/L, lymphocytes at 1.4 x 10^9^/L. Liver function tests and electrolytes are all within the normal range.

His other co-morbidities included dementia, which progressed and resolved from time to time after starting treatment with HIV protease inhibitor triple therapy in 1996, complications with anti-retroviral medications such as marked wasting syndrome, Addison's disease, peripheral neuropathy, osteoporosis, anal dysplasia, warts, fissures, rectal prolapse, chronic obstructive pulmonary disease (COPD), and bronchiectasis due to longstanding smoking.

His case was discussed in our tumor board rounds. His HIV status and low CD4 count were considered relative contraindications for high-dose CMT. Previous publication from 1999 reported poor results of CMT for HIV-positive AC if CD4 is less than 200 mm^-3^. Most of the patients required either hospitalization or colostomy [4]. He clearly stated that he would rather die from the cancer if he would require a colostomy.

**Figure 2:**
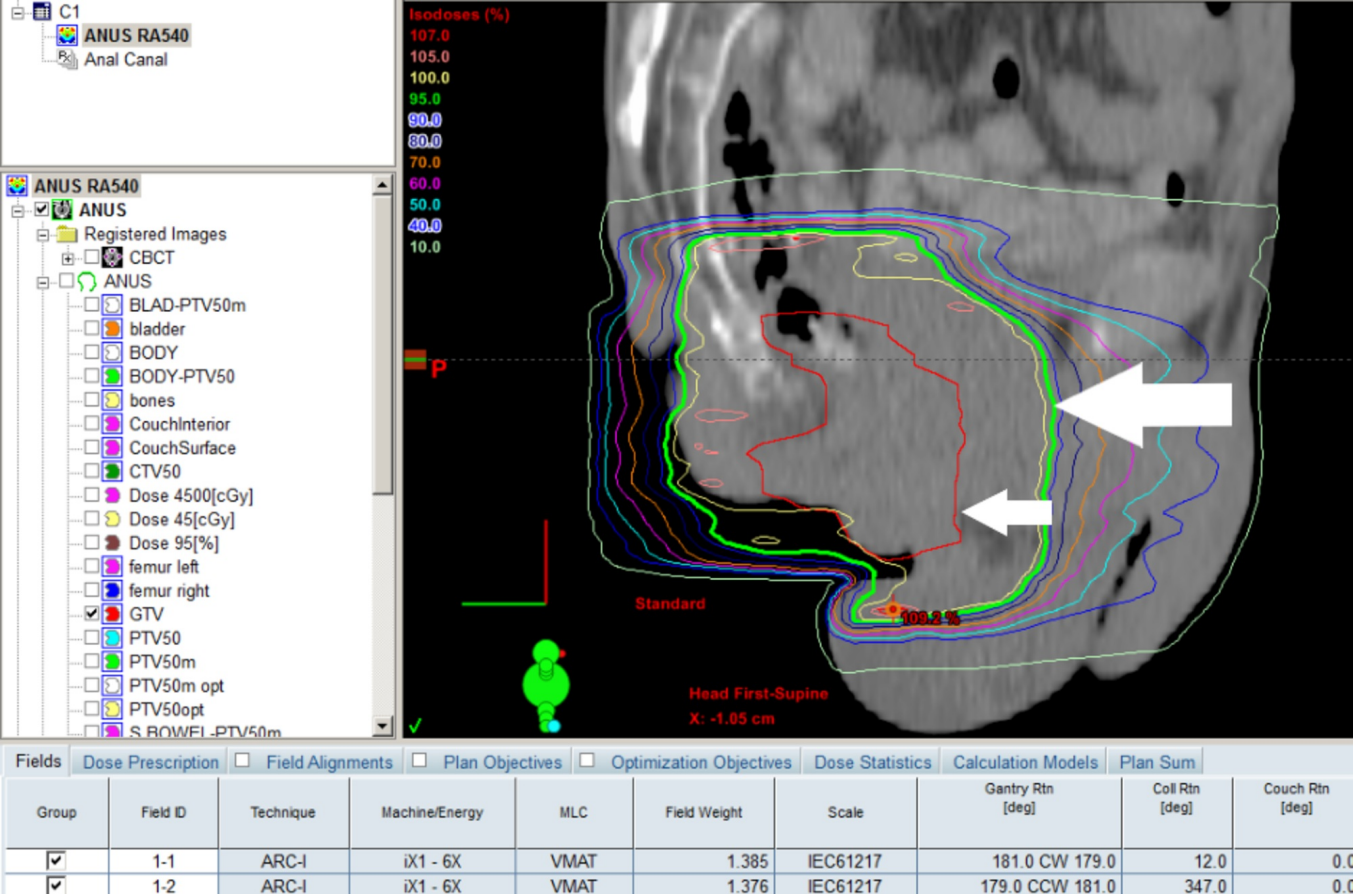
VMAT plan sample sagittal view showing GTV in red (small arrow) and 95% dose curve in green (big arrow) VMAT, volumetric modulated arc therapy; GTV, gross tumor volume

He was not considered a good candidate for standard CMT. Eventually, he received EBRT alone to the primary cancer, 50-Gy in 25 fractions over five weeks, with the last dose on April 11, 2014. He did not have any treatment for the inguinal or pelvic lymph nodes in an effort to avoid severe toxicities. We used Eclipse treatment planning system to plan Rapid Arc VMAT (Figure 2).

**Figure 3:**
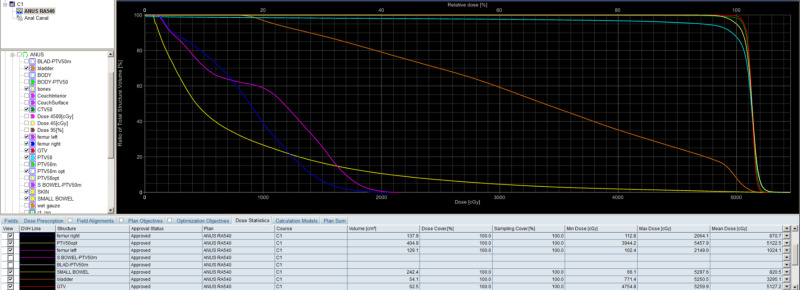
Cumulative dose volume histogram (DVH)

He had a CT simulation with bowel contrast, empty rectum and Vac-Loc immobilization as in our standard protocol. The gross tumor volume (GTV) was 62.5-ml, clinical target volume (CTV) was GTV plus 1-cm expansion to account for microscopic disease, and planning target volume (PTV) was CTV plus 1-cm expansion to account for tumor movement, patient movement, daily setup error, etc. We used 1-cm bolus on peri-anal skin. The organs-at-risk (OAR) included the small bowels, bladder, and femoral head. All normal tissue tolerance dose constraints were met on the dose volume histogram (DVH) (Figure 3).

**Figure 4:**
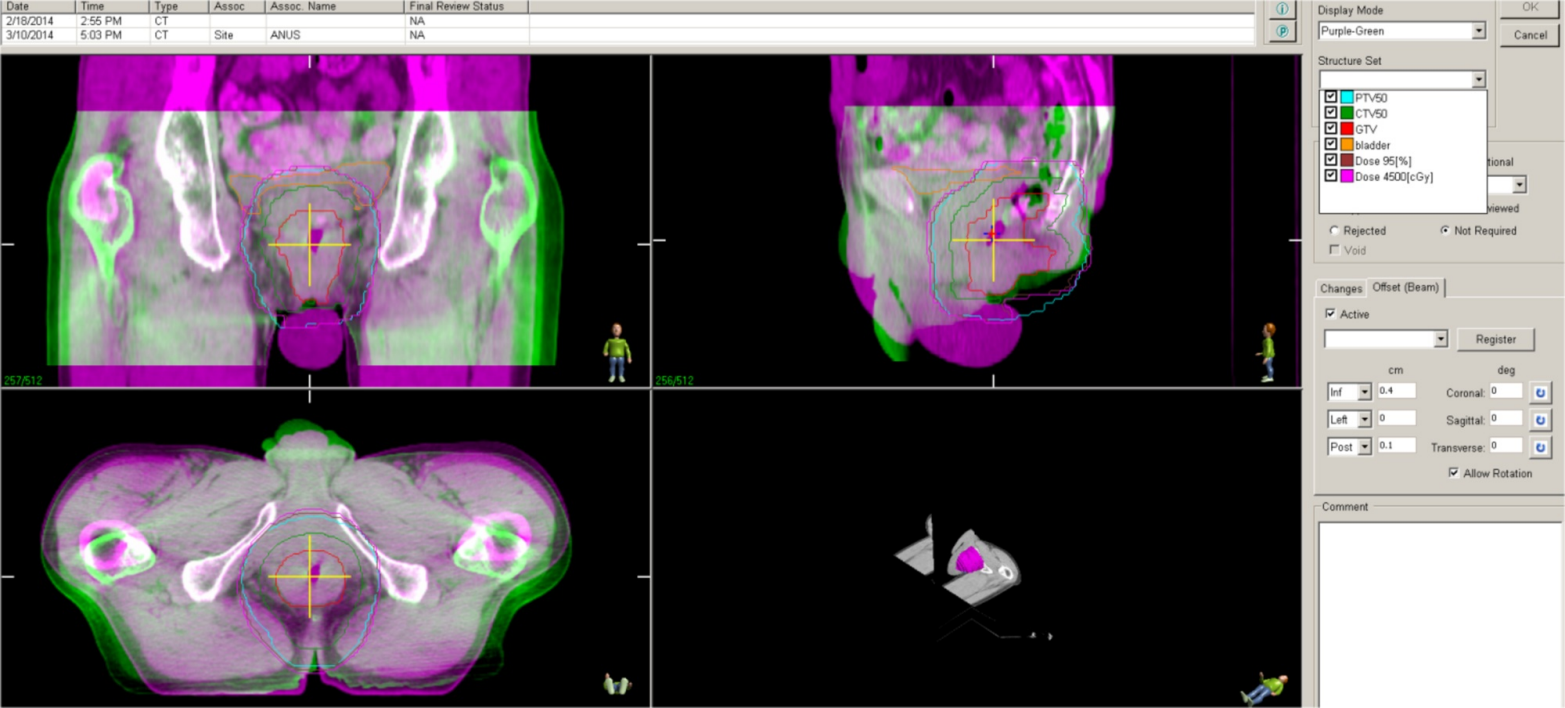
Daily CBCT in green overlapping with planning CT in purple Note Beam Offset of only 0.4 cm, 0 cm and 0.1 cm to the inferior, left and posterior, respectively, which will be auto-matched to pelvic bone. CBCT, cone beam computed tomography; CT, computed tomography; PTV50, planning target volume to receive 50Gy; CTV50, clinical target volume to receive 50Gy; GTV, gross tumor volume

He had image-guided radiation therapy (IGRT) with daily cone-beam CT (CBCT) guidance. CBCT was matched to pelvic bone with manual assessment of CTV and PTV (Figure 4). The treatment unit was Varian Clinac iX Linear Accelerator. We used 6MV photons and 540-degree arcs. Average machine time was only six minutes, from the time of CBCT to the completion of VMAT delivery, in contrast to 15 minutes in our standard multiple field intensity-modulated radiation therapy (IMRT). This helped with patient comfort and encouraged compliance.

**Figure 5:**
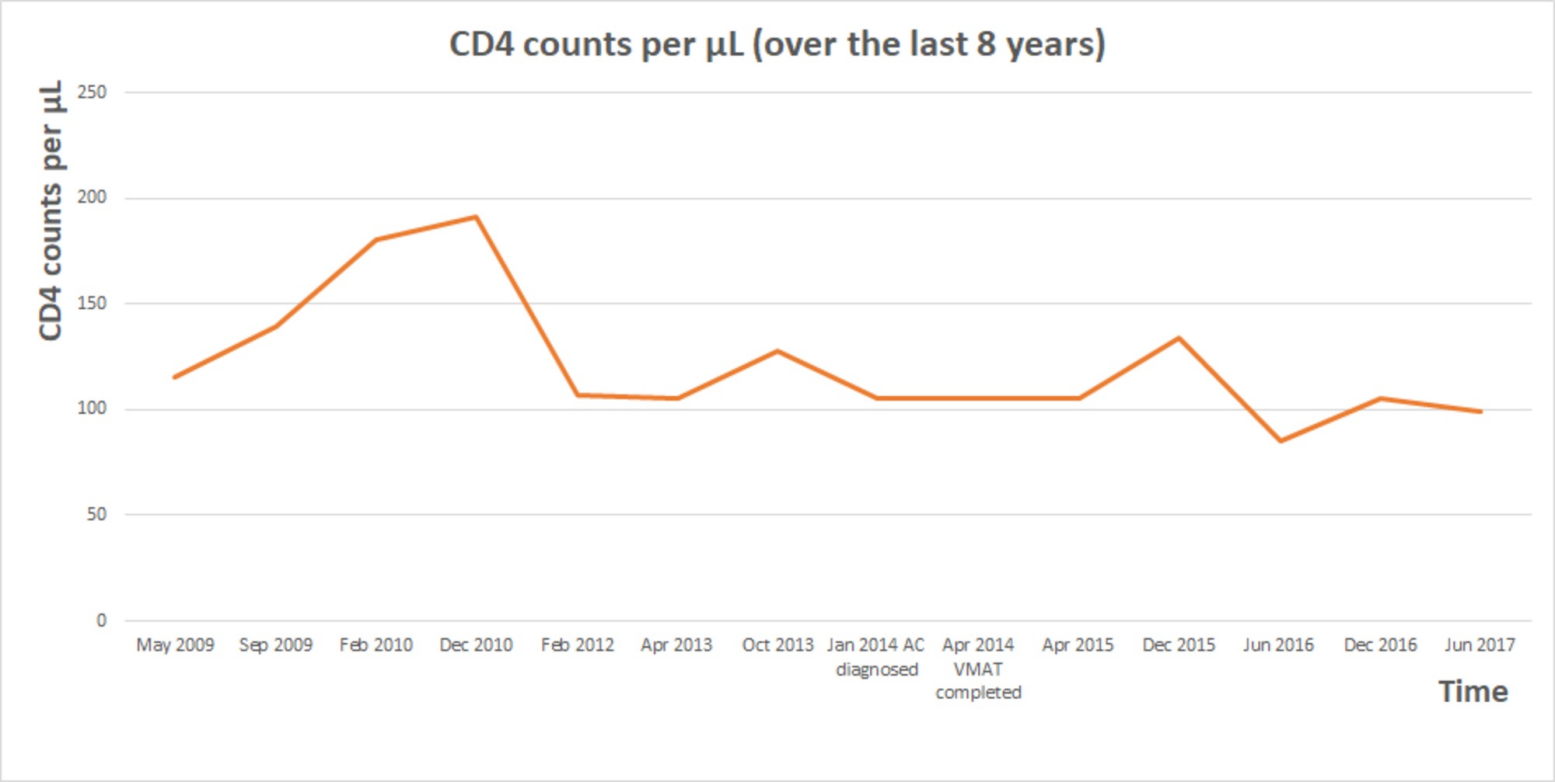
T cell CD4 counts over eight years AC, anal canal cancer; VMAT, volumetric modulated arc therapy

He had some grade 2 acute toxicities including anal pains and moist desquamation in perineal/peri-anal skin, which were treated accordingly and subsequently settled down. He continued HAART for AIDS and treatment for newly diagnosed hepatitis C. His CD4 cell count remained stable at 105, 134, 85, 105 and 99 mm^-3^, at 12, 20, 26, 32 and 38 months after the completion of VMAT, respectively (Figure 5).

**Figure 6:**
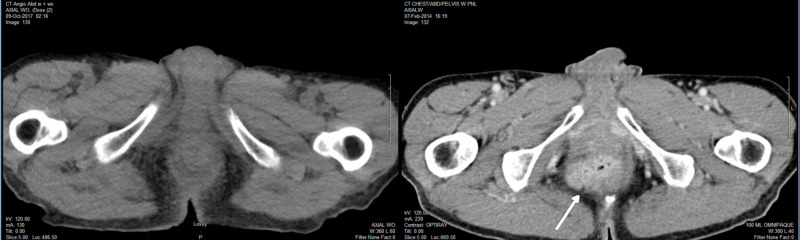
Last follow-up CT scan showing no local recurrence on the left, versus the large tumor (white arrow) on the pre-treatment CT on the right CT, computed tomography

On the last follow-up, he continued to have occasional fecal incontinence, but he was managing it well. He did not notice any change in his bowel habits, rectal bleeding or pain. There was no recurrent disease on digital rectal exam (DRE). His last CT scan did not show any recurrent cancer, enlarged lymphadenopathy or distant metastases (Figure 6). His AC was considered having been cured, so he was discharged from the cancer clinic but continued to be followed by palliative care physicians for his dementia. His last colonoscopy only showed mild radiation proctitis without any cancer or bleeding at 42 months after VMAT.

Unfortunately, his dementia deteriorated and he died from a combination of hepatic encephalopathy, aspiration pneumonia and sepsis 47 months after the diagnosis of ACSCC.

## Discussion

The risk of AC is not declining with increasing usage of HAART [2,3,12-13]. Bower et al. reported a large cohort of 8640 HIV-seropositive individuals. The incidence of invasive AC is 120 times higher than in the age- and gender-matched general population. The incidence of invasive AC in the HIV cohort was 35 (95% confidence interval CI: 15-72) per 100,000 patient-years of follow-up in the pre-HAART era (1984-1995) and 92 (95% CI: 52-149) per 100,000 patient-years of follow-up in the post-HAART era (1996-2003) (P > 0.05). These give a relative risk of 67 and 176 in the pre- and post-HAART eras, respectively, compared with the general population [12]. Some early studies suggested that CMT might be too toxic in patients with CD4 < 200 mm^-3^ at the time of AC diagnosis [4,14]. Thus, CMT was considered a relative contraindication for these patients. For reference, the CD4 count of an uninfected adult ranges from 500 to 1,600 cells mm^-3^.

Holland and Swift reported seven AC patients with HIV all required CMT treatment breaks of a mean duration of 21.7 days. Three (43%) patients required hospitalization, four (57%) patients required chemotherapy dose reduction. They concluded that these patients have increased toxic reactions to CMT, thus treatment must be individually tailored [5].

Bottomley et al. reported the outcome of six HIV-positive AC patients. Durable complete responses with acceptable toxicity occurred in two patients with moderate immunosuppression and Stage I-II tumors treated with CMT (45-Gy in 25 fractions). One patient treated with EBRT alone (60-Gy in 30 fractions in two phases) also had a complete response. Two patients, one with Stage III tumor and the other with pre-existing AIDS, died within six months of treatment. The authors supported the use of CMT in selected patients, but cautioned that the appropriate treatment of patients with more advanced tumors and/or advanced HIV infection is uncertain [10].

The toxicity of CMT with HAART in some recent series might have diminished. In the largest study with 60 cases, Alfa-Wali et al. reported five-year overall survival (OS) of 38% and 68% for the pre- and post-HAART eras, respectively [15]. For patients treated with CMT, the median CD4 fell by half during the first three months of therapy (P < 0.0001) and remained below pre-CMT levels throughout a year of follow-up. Their patient cohort was young (median age 44 years) and healthier, with 78% of the HAART patients having an undetectable viral load. The median CD4 at diagnosis of AC for the entire cohort was 305 mm^-3^ (range: 16-1252 mm^-3^). By comparison, the randomized United Kingdom Co-ordinating Committee on Cancer Research ACT I trial included 292 HIV-negative patients treated with CMT and the five-year OS was only 58% [16]. This indicates that the Alfa-Wali study’s patient cohort might not be representative of AC with positive HIV.

Our institutional AC protocol was based on RTOG 98-11 study of 644 HIV-negative patients randomized to neo-adjuvant and concurrent cisplatin with 5-FU versus concurrent mitomycin-C and 5-FU, both with same dose EBRT 45-59 Gy. Their five-year OS (70% versus 75%) and disease-free survival (54% versus 60%) was similar to other studies. Cumulative colostomy rate was reasonable, that is, 10% in mitomycin arm and 19% in cisplatin arm [17]. Since there are not enough AC patients with HIV-positivity to conduct an RCT, we have to assume that they have a much worse outcome and colostomy rate than that of the RTOG study, according to limited retrospective studies and case reports [4,5,10,14,15,18,19]. It should be noted that none of these studies used VMAT, as this new technology just became available in the recent decade.

For HIV-negative AC patients, National Comprehensive Cancer Network (NCCN) Clinical Practice Guidelines in Oncology recommended CMT including IMRT or VMAT at least 30.6-Gy to low risk inguinal lymph node PTV and 45-Gy to high-risk pelvic lymph node PTV, followed by an additional boost of 5.4-14.4 Gy to residual T1-2, T3-4 lesions, or N1 lesions, all in 1.8-2 Gy daily fractions for a total dose of 50.4-59.4 Gy [20]. The recommendation for HIV-positive AC patients is less clear. There was no published report that suggested pelvic irradiation can be safely avoided.

Alfa-Wali et al. concluded that CMT is associated with significant prolonged CD4 suppression that may contribute to late death of patients in remission. Post-HAART patients had a median CD4 of 332 mm^-3^ which dropped to 132 mm^-3^ at three months and 200 mm^-3^ at 12 months after CMT, while the other eight pre-HAART patients had median CD4 of 209 mm^-3^ which dropped to 116 mm^-3^ at three months and 85 mm^-3^ at 12 months [15]. We would rather consider our patient to be similar to the pre-HAART cases due to his noncompliance to HAART. He would likely have had a poor tolerance or even a fatal response to CMT due to the toxicity. More importantly, he was very keen on his QoL and refused any treatment that could potentially lead to a diverting colostomy. He agreed with VMAT to the primary tumor only, without radiation to the inguinal area or pelvis and without chemotherapy, after informed consent.

At the time when he passed away due to dementia-related sepsis, he had no evidence of local failure or distant metastasis. We kept his QoL without colostomy for 44 months since the completion of VMAT. This case could be one of the longest AIDS survivors (PubMed search) successfully treated with VMAT alone for locally advanced ACSCC ever reported in the literature.

## Conclusions

Our case suggests that VMAT to the primary tumor alone up to 50-Gy in 25 fractions over five weeks, without treating the inguinal or pelvic lymph nodes, can be a safe and effective treatment for node-negative locally advanced ACSCC patients with AIDS even when HIV viral load is over one million and CD4 cell count is very low around 100 mm^-3^. Long-term local control can be achieved with reasonably tolerated acute and late toxicities. Radical dose, instead of palliative dose, VMAT might be considered for selected patients of this group when CMT is not desirable due to the concerns of severe side effects.
